# Supplementary material for: Caenorhabditis elegans foraging patterns follow a simple rule of thumb
Source: Commun Biol. 2023 Aug 14;6:841. doi: 10.1038/s42003-023-05220-3 (PMC10425387; doi:10.1038/s42003-023-05220-3)
Supplement: Supplementary file 2 — Supplementary Information [file 42003_2023_5220_MOESM2_ESM.pdf]

Supplementary Information

***Caenorhabditis elegans* foraging patterns follow a simple rule of thumb**

Gabriel Madirolas*[1], Alid Al-Asmar*[1], Lydia Gaouar[1], Leslie Marie-Louise[1], Andrea Garza-Enriquez[1], Valentina Rodríguez-Rada[1], Mikail Khona[2], Martina Dal Bello[2], Christoph Ratzke[2,3], Jeff Gore[2], Alfonso Pérez-Escudero[1]

[1] Research Centre on Animal Cognition (CRCA), Centre for Integrative Biology (CBI), Toulouse University, CNRS, UPS, Toulouse 31062, France.

[2] Physics of Living Systems Group, Department of Physics, Massachusetts Institute of Technology, United States

[3] Interfaculty Institute for Microbiology and Infection Medicine Tübingen (IMIT), Cluster of Excellence EXC 2124 “Controlling Microbes to Fight Infections” (CMFI), University of Tübingen, Calwerstrasse 7/1, 72076 Tübingen

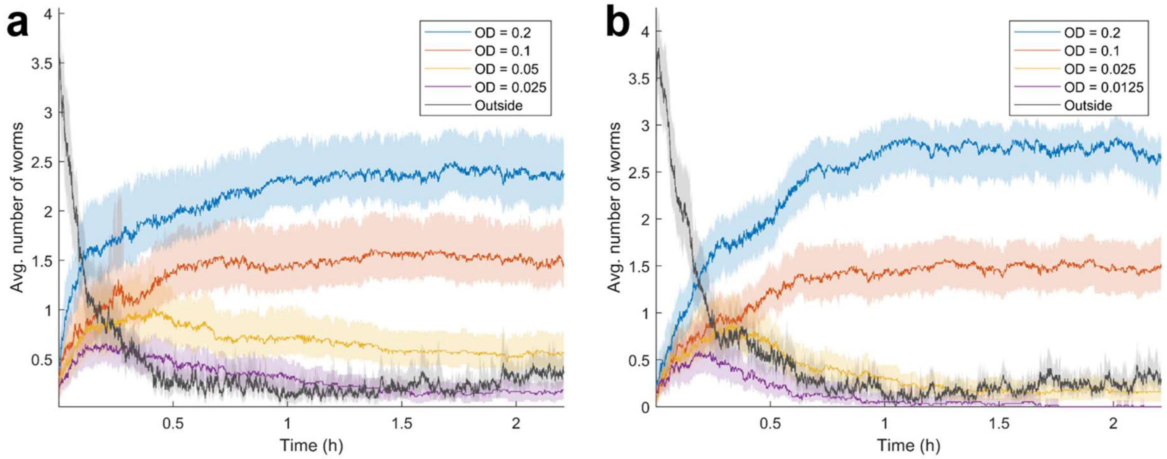

Supplementary Figure 1. Dynamics of *C. elegans* foraging experiments. (A) Average number of worms in each patch (or outside the patches) as a function of time for two experiments with 4 food patches. Averaged over 58 videos, with 299 worms in total. Shaded areas show the 95% confidence intervals, computed via bootstrapping. Food patches were composed of *E. coli* OP50 with densities 0.2, 0.1, 0.05, and 0.025 (measured as Optical Density). Food patches were located at 1.2 cm of the center of the plate, forming a square. Worms started at the center of the square. All other experimental details were identical as those in the main text, except that the plates did not contain novobiocin (these experiments were performed with *E. coli* OP50 only, and this species' growth is effectively arrested by chloramphenicol alone). **(B)** Same as A, but for patch densities 0.2, 0.1, 0.025 and 0.0125 (measured as Optical Density), and averaged over 56 videos, with 266 worms in total.

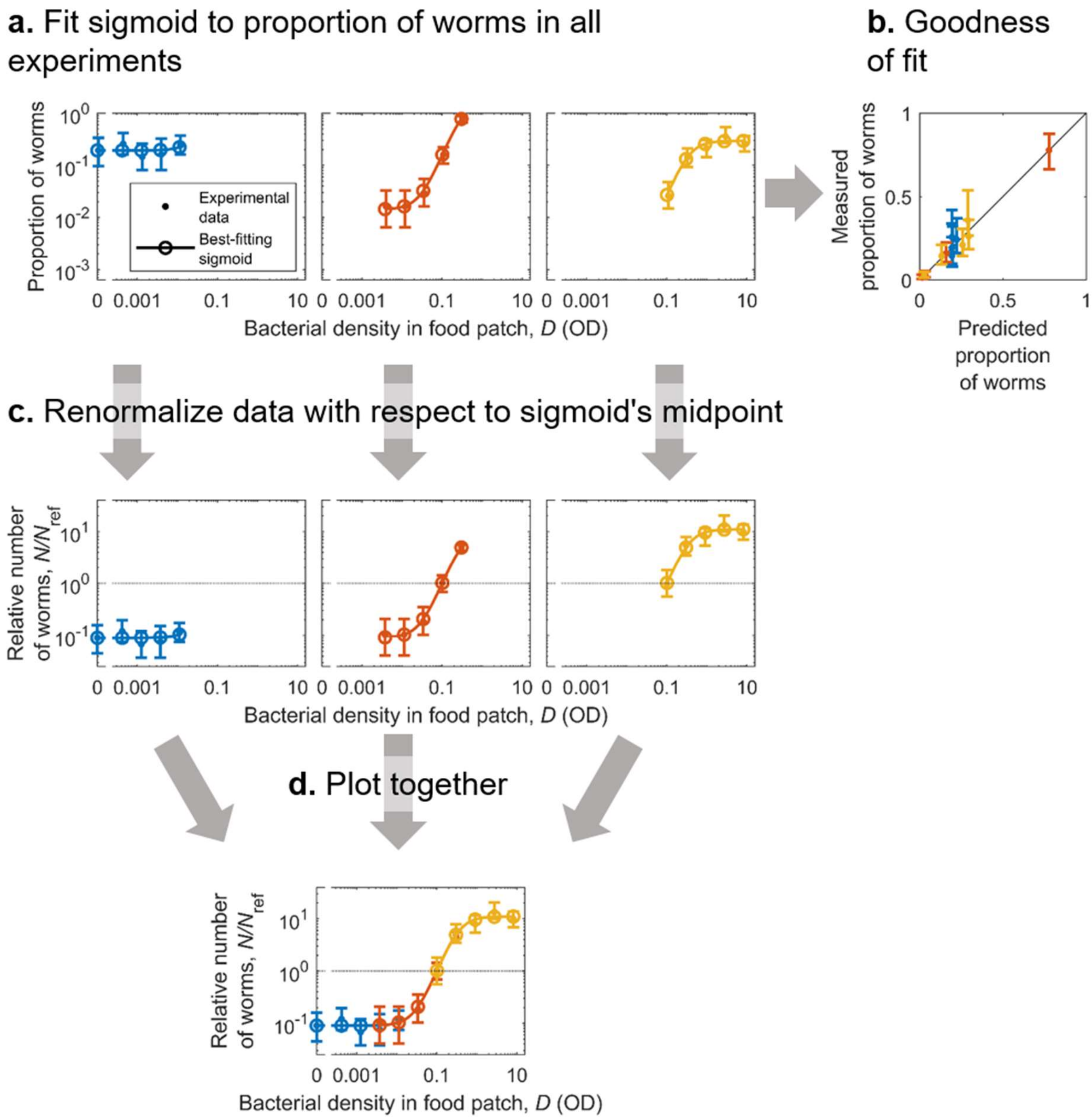

Supplementary Figure 2: Illustration of data processing and normalization. Each complete sigmoid comes from several experiments, each of them covering part of the density range (in this case, 3 experiments with 5 food patches each; each of the three experiments is shown in one column and in a different color). **(a)** Proportion of worms present at each food patch in each experiment (dots with errorbars). These proportions add up to 1 for each experiment. Lines and circles: Best-fitting sigmoid. The three experiments (i.e. three columns) are described by the same sigmoid (**Equation 1** with a single set of parameters), but the sigmoid is normalized for each experiment using **Equation 2**. **(b)** Measured proportions versus predicted proportions, for all food patches of the three experiments. The black line corresponds to a perfect prediction. **(c)** Normalized number of worms, N/N_{ref}, where N is the number of worms in each food patch, and N_{ref} is the number of worms in a virtual reference patch (**Equation 4**). **(d)** Same as (c), but with all data in the same plot. See **Supplementary Data 1** for the data and computer code that generate this figure.

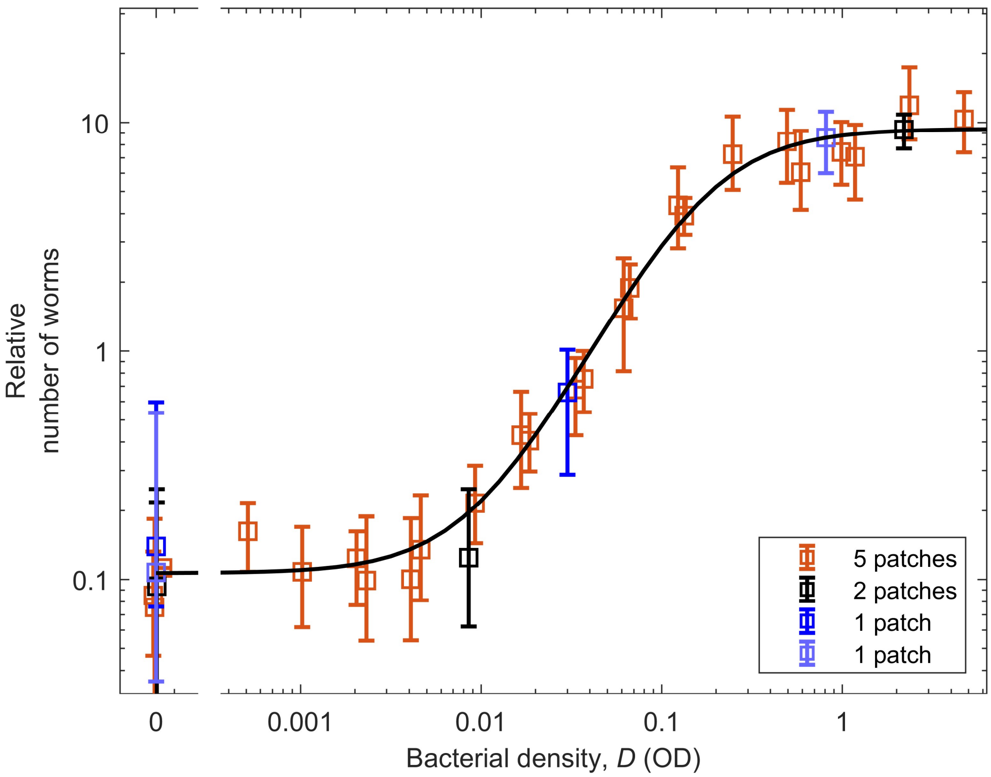

Supplementary Figure 3: Validation of the normalization procedure. Relative number of worms found at each food patch, as a function of bacterial density in the food patch (*D*). Squares: Experimental data for *E. coli* OP50; errorbars show the 95% confidence interval, computed via bootstrapping. Line: Fitted sigmoid, following **Equation 1 in Methods**. The validation was performed as follows: We first studied 5 experimental conditions, each of them with 5 food patches, collectively covering the whole density range (orange points). We applied our normalization procedure as described in the methods to these 5 dataset, obtaining the sigmoid (black line). Then, we performed three separate experiments, one of them with only two food patches of densities 0.008 and 2.16 (black squares). Finally, we performed another two experiments with a single food patch, with densities 0.03 (dark blue squares) and 0.81 (light blue squares). We used the same geometry for all experiments, so for these control experiments the densities at the five corners of the pentagon were [0, 0, 0, 0.008, 2.16], [0, 0, 0, 0, 0.03], [0, 0, 0, 0, 0.81]. Therefore, the two-patch experiment has three datapoints in the figure (the two patches plus zero density), and each of the one-patch experiments have two datapoints (the patch plus zero density). The results from each of these experiments were multiplied times a renormalization constant, to transform from absolute to relative number of worms. Note that this normalization cannot change the ratio between the different datapoints within each experiment. These ratios agreed exactly with the ones predicted by the sigmoid (black and blue squares). See **Supplementary Data 1** for the data and computer code that generate this figure.

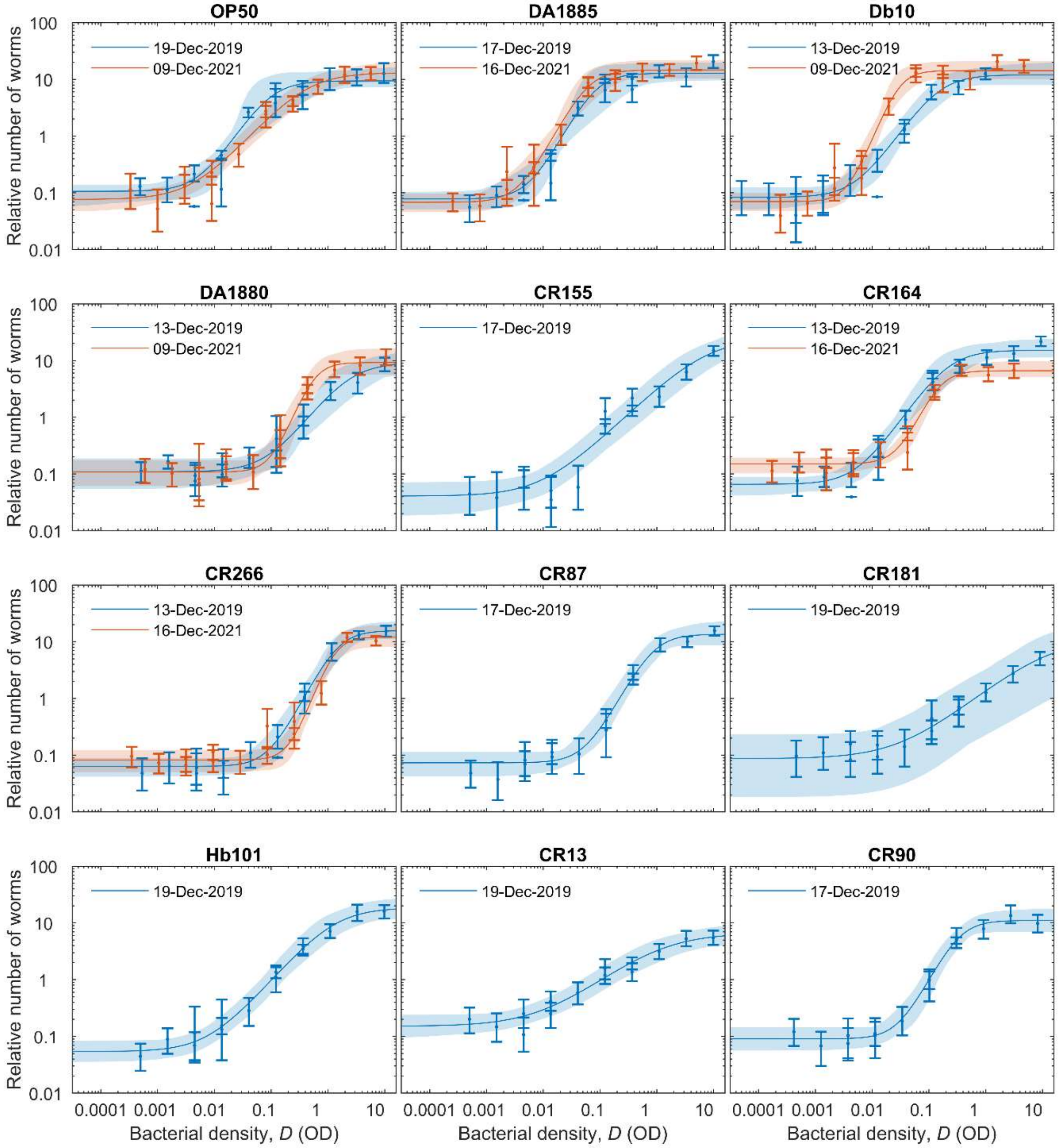

Supplementary Figure 4: Sigmoids for all strains. Relative number of worms found at each food patch, as a function of bacterial density in the food patch (*D,* measured in Optical Density, OD). Dots: Experimental data; errorbars show the 95% confidence interval. Lines: Best-fitting sigmoid. Semitransparent areas: 95% confidence interval for the fitted sigmoids. Six strains were measured twice, and the lockdown due to the COVID-19 pandemic imposed a two-year gap between them, a period during which the laboratory moved to a new building. As a result, both replicates were performed in different conditions: 2019 experiments were performed in an incubator and the main experimenter was LG, while 2021 experiments were performed in an environmental room and the main experimenter was AAA. All other experimental parameters were kept as equal as possible. We chose to show these results rather than repeating the experiments in the same conditions to highlight the robustness of our results: While two strains (Db10 and CR164) show significant differences across the two replicates, we found remarkable reproducibility given the experimental differences. Results shown in **Figures 1** and **3** of the main text correspond to the 2019 experiments. See **Supplementary Data 1** for the data and computer code that generate this figure.

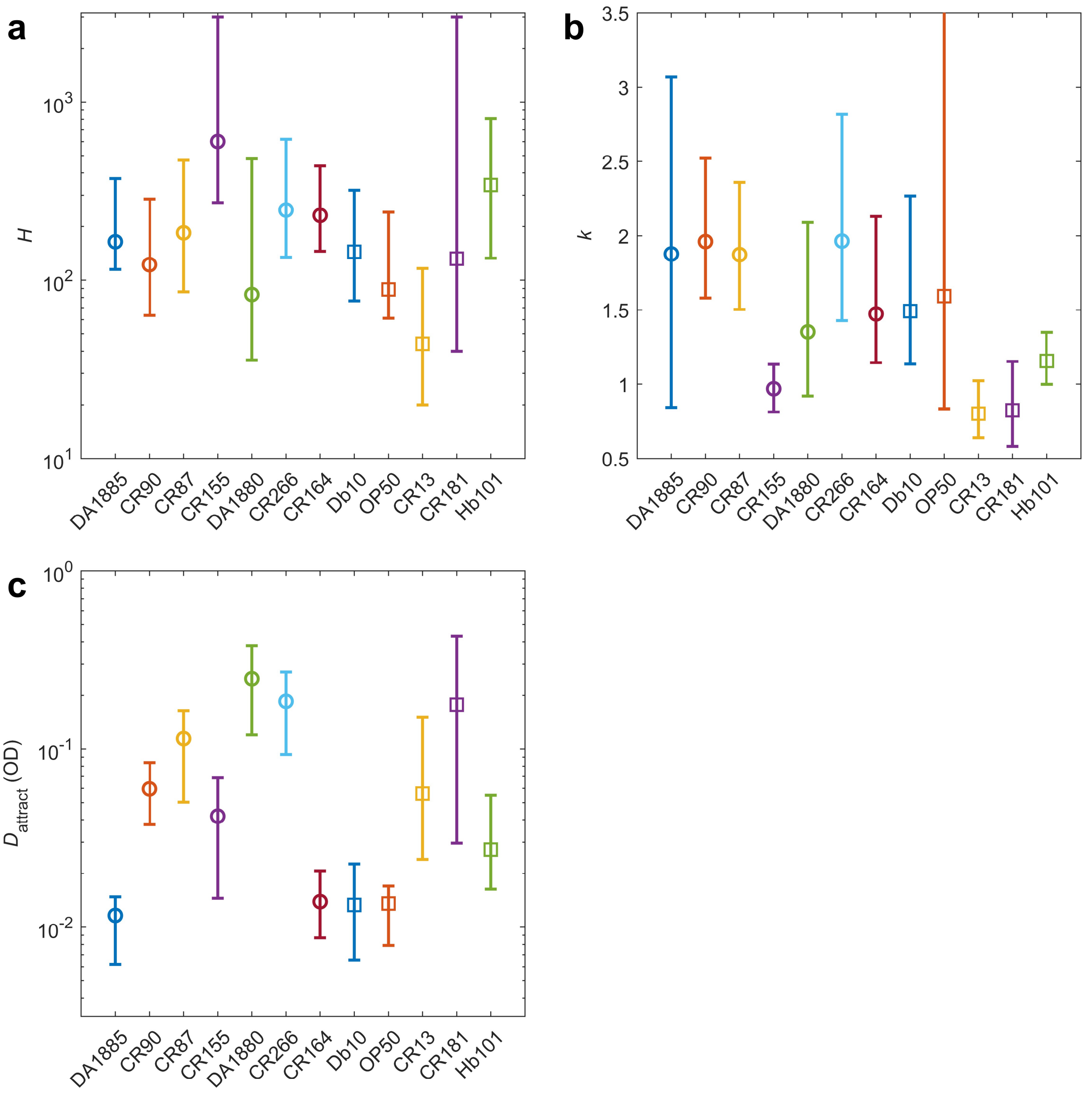

Supplementary Figure 5: Sigmoid parameters for all strains. a. Best-fitting value of H for each bacterial strain. **b.** Best-fitting value of k for each bacterial strain. **c.** Best-fitting value of the attraction density (D_{attract}) for each bacterial strain. All errorbars show the 95% confidence interval, calculated via bootstrapping. See **Supplementary Data 1** for the data and computer code that generate this figure.

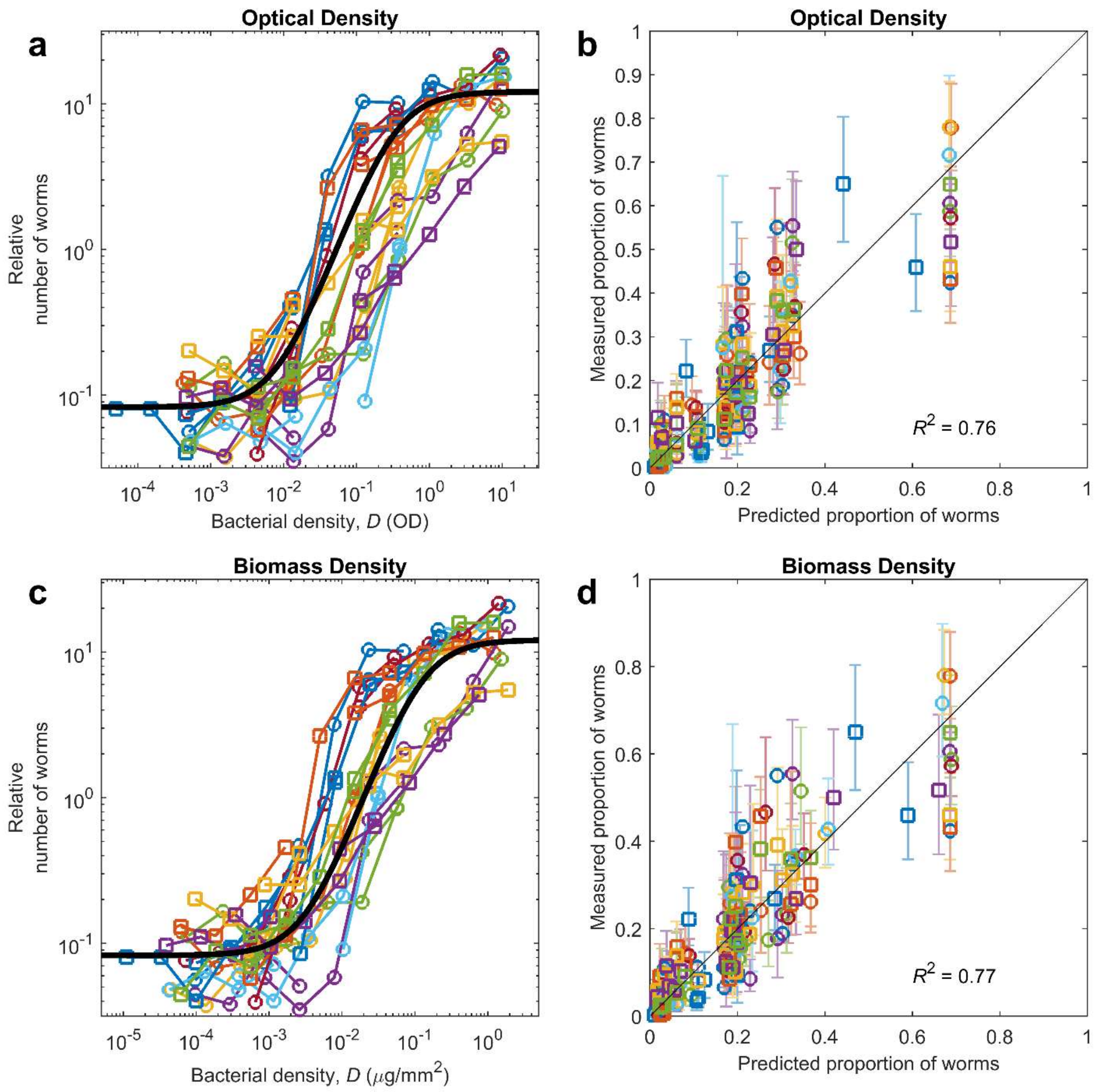

Supplementary Figure 6. Comparison of results when using different measurements of density as the single environmental variable. Color and shape of markers identify bacterial strains (see legend in **Figure 1**). **a.** Relative number of worms found at each food patch, as a function of bacterial density (measured in OD) in the food patch. Points coming from the same experimental condition are linked by lines. Black line: Sigmoid, fitted to all strains. **b.** Measured proportion of worms in each food patch, versus proportion predicted by the sigmoid in (a). All errorbars show the 95% confidence interval, computed via bootstrapping. **c.** Same as (a), but with bacterial density measured as biomass density. **d.** Same as (b), but with using biomass density to perform the prediction.

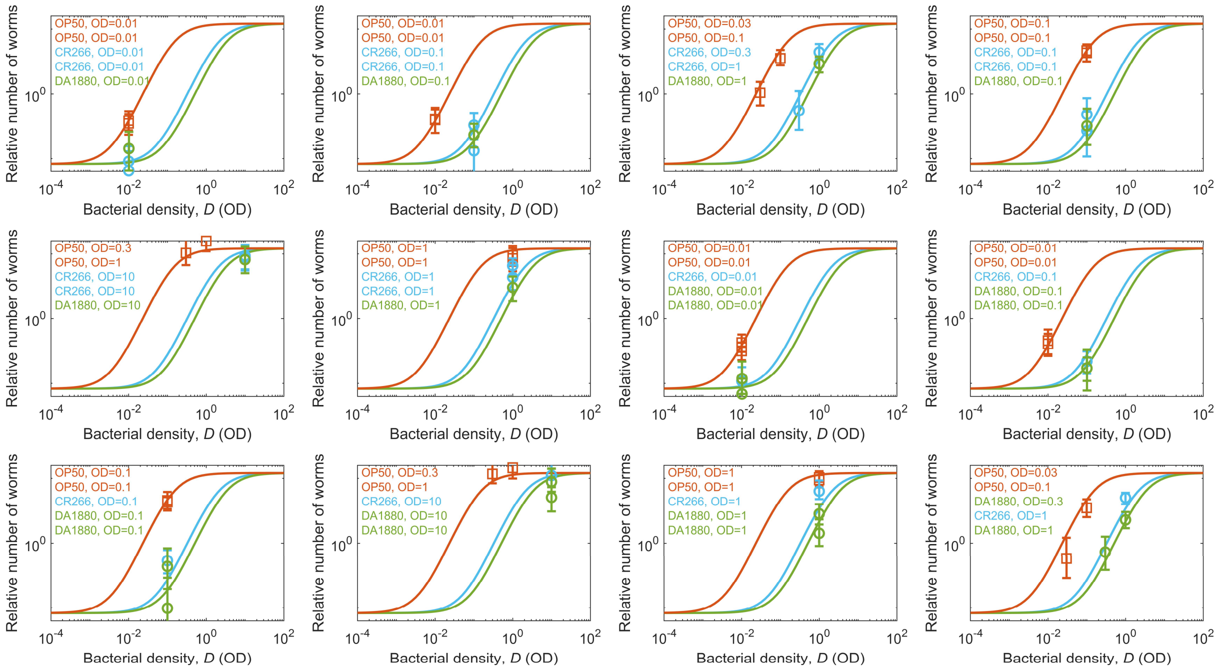

Supplementary Figure 7: Results in environments with food patches of different species. Relative number of worms as a function of bacterial density (measured as optical density, OD). Each box shows a different 5-patch condition, with the bacterial strains and densities shown in the legend. Lines: Sigmoidal model, with parameters $H = 146$, $k = 1.4$, $D_{\text{attract, OP50}} = 0.01$, $D_{\text{attract, CR266}} = 0.15$, $D_{\text{attract, DA1880}} = 0.23$. Dots: Experimental results. All error bars show 95% confidence intervals, computed via bootstrapping. Note that here we have chosen to represent density as Optical Density, so the effect of D_{attract} is to shift the sigmoid for each bacterial strain. In contrast, **Figure 4b** of the main text uses effective density (D/D_{attract}), so the the effect of D_{attract} is to shift the experimental data, so that they all match with the same sigmoid. See **Supplementary Data 1** for the data and computer code that generate this figure.

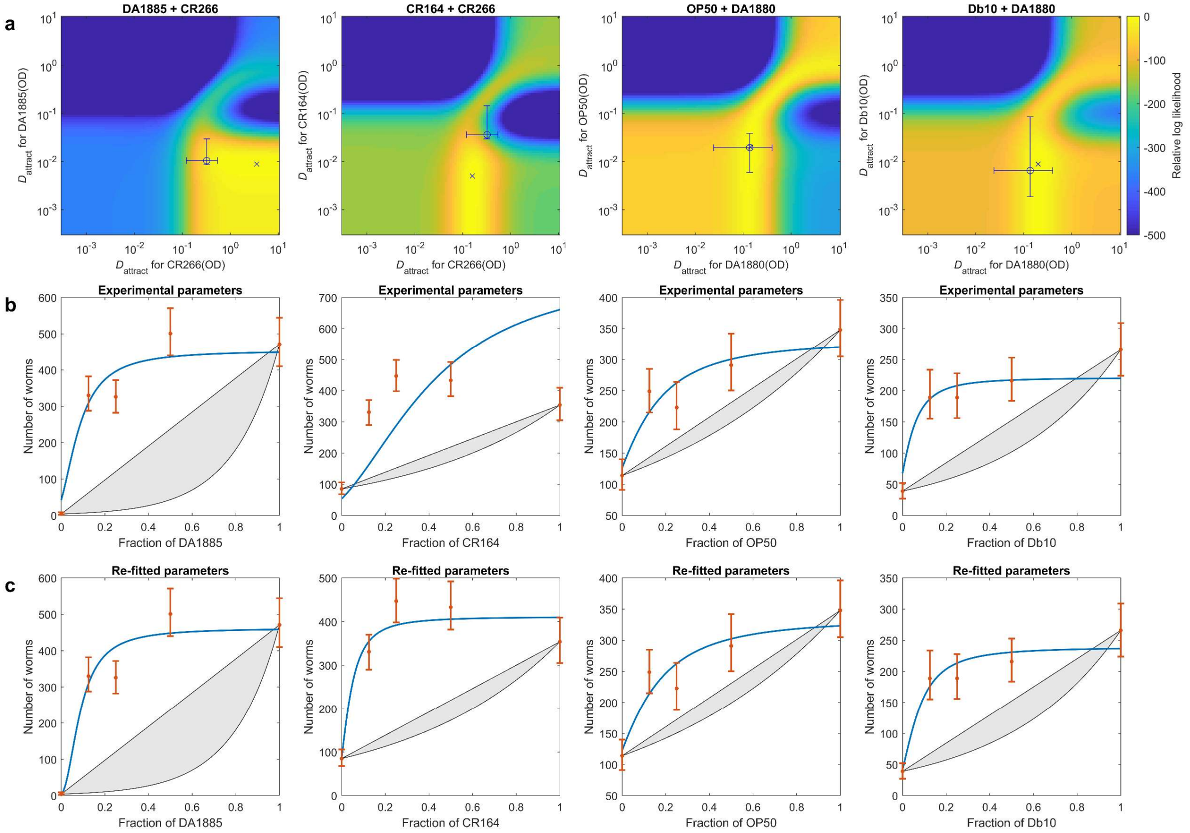

Supplementary Figure 8: Results from mixed drops. Each column corresponds to one pair of strains. **a.** Goodness of fit of our prediction to the experimental data, for each possible value of D_{attract} for each strain (hotter color means better fit). Circle with errorbars: Experimental D_{attract}, obtained from the sigmoidal fit to an independent experiment (orange sigmoids in **Figure S4**). Cross: Values of D_{attract} that best reproduce the mixed-drop experiments. In the last two pairs, the experimental values are very close to the best-fitting one. In the first pair the optimal value is far from the experimental one, but the experimental values are still on the region of excellent fit (yellow region). In the second pair the fit is not good: The experimental values fall outside the good fitting region. This pair involves strain CR164, which also gave a sigmoid significantly different to the one originally measured (see **Figure S4**). Therefore, it's likely that experimental variability was responsible for this mismatch, but we cannot exclude other factors. **b.** Number of worms at each food patch, as a fraction of one of the strains. Dots: Experimental results (errorbars show the 95% confidence interval). Red line: Prediction from our model, using the experimentally measured D_{attract} (circle in box A). Gray patch: Prediction of the null model. **c.** Same as B, but prediction uses the best-fitting values of D_{attract} (cross in box A). See **Supplementary Data 1** for the data and computer code that generate this figure.

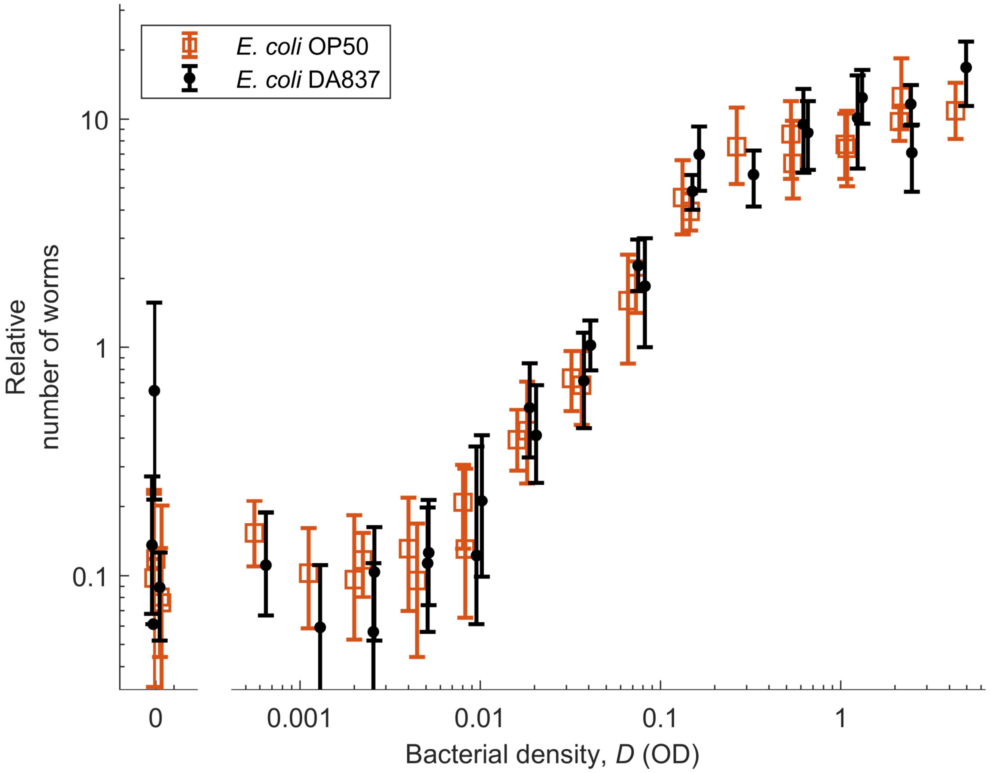

Supplementary Figure 9: *C. elegans* shows the same response to OP50 and DA837. Relative number of worms found at each food patch, as a function of bacterial density in the food patch (*D*). Squares: *E. coli* OP50. Dots: *E. coli* DA837. Errorbars show the 95% confidence interval, computed via bootstrapping. See **Supplementary Data 1** for the data and computer code that generate this figure.

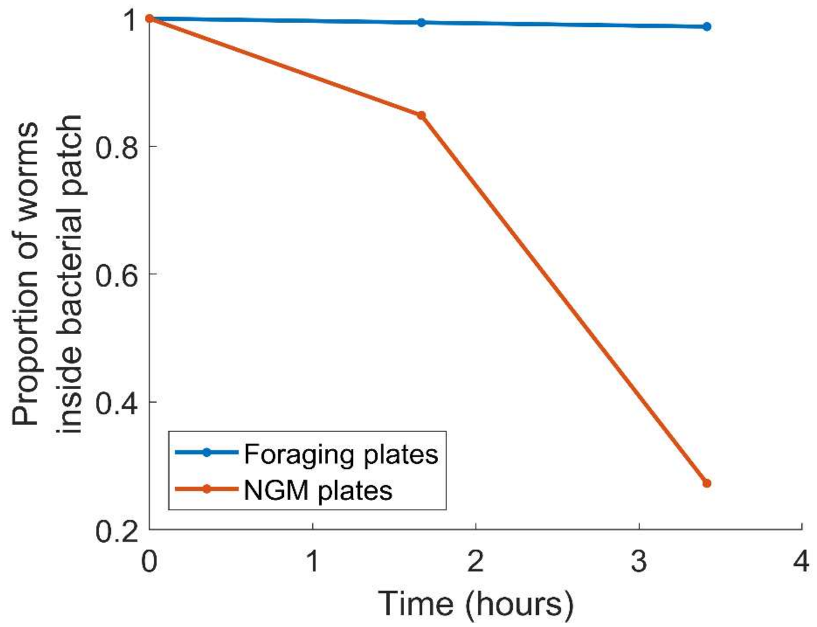

Supplementary Figure 10: Avoidance of *Serratia marcescens* requires active bacterial growth. We placed a 40 microliter drop of saturated overnight culture of *S. marcescens* (Db10) at the middle of either an NGM plate (where bacteria can grow) or a foraging plate (where bacteria cannot grow due to lack of nutrients and presence of bacteriostatic antibiotics). The next day, we placed around 30 young adult worms (48-hour old) at the center of the food patch. The figure shows the proportion of worms that remained inside the food patch as a function of time, for both treatments. These results are consistent with previous studies, which found strong avoidance of *S. marcescens* on NGM plates.[41] See **Supplementary Data 1** for the data and computer code that generate this figure.

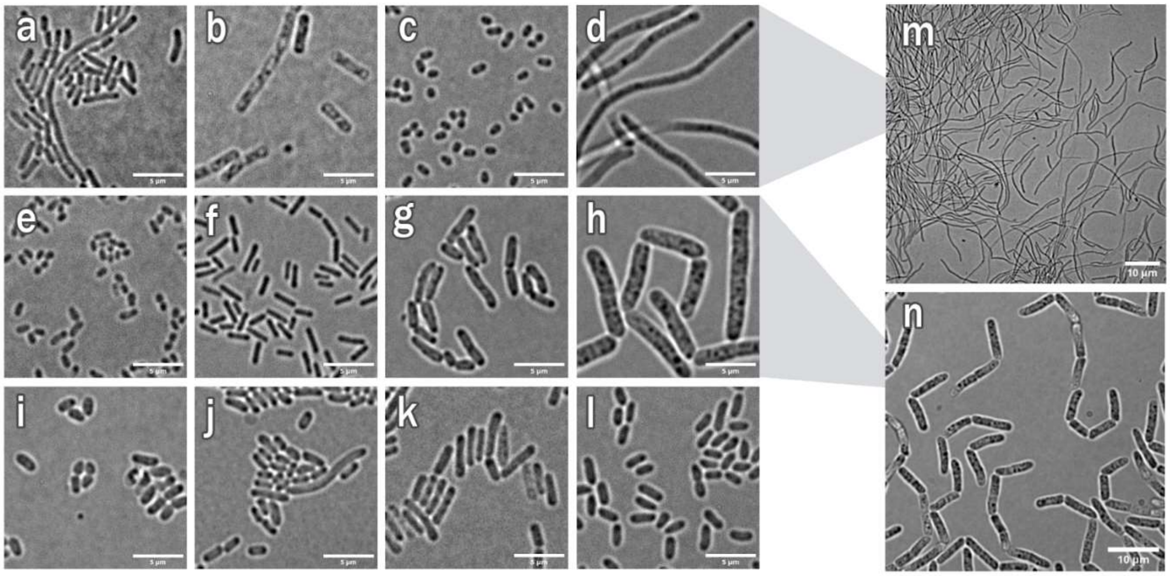

Supplementary Figure 11: Images of all bacterial strains. Optical microscope images of all our bacterial strains. **(a)** OP50, **(b)** DA1885, **(c)** Db10, **(d)** DA1880, **(e)** CR155, **(f)** CR164, **(g)** CR266, **(h)** CR87, **(i)** CR181, **(j)** Hb101, **(k)** CR13, **(l)** CR90. **(m)** DA1880 (lower magnification, showing full filaments filaments) and **(n)** CR87 (lower magnification, showing chains of bacteria).

Figure	Species	Number of plates per point			
		Average	Min	Max	
Figure 1b	OP50	27.8	25	30	
Figure 1d-g	DA1885	26.0	21	32	
Figure 1d-g	CR90	29.3	27	31	
Figure 1d-g	CR87	31.3	30	32	
Figure 1d-g	CR155	26.7	26	27	
Figure 1d-g	DA1880	24.0	11	31	
Figure 1d-g	CR266	31.7	31	32	
Figure 1d-g	CR164	28.0	25	32	
Figure 1d-g	Db10	32.0	32	32	
Figure 1d-g	OP50	25.3	22	31	
Figure 1d-g	CR13	31.7	31	32	
Figure 1d-g	CR181	30.7	30	31	
Figure 1d-g	Hb101	30.3	28	32	
Figure 2b-c	DA1885	5.8	2	8	
Figure 2c	CR90	5.0	1	8	
Figure 2c	CR155	5.4	3	7	
Figure 2c	DA1880	4.2	2	7	
Figure 2c	CR266	5.2	3	7	
Figure 2c	CR164	4.4	1	7	
Figure 2c	Db10	5.3	2	8	
Figure 2c	OP50	3.9	0	7	
Figure 2c	CR13	4.0	1	5	
Figure 2c	CR181	3.7	2	7	
Figure 2c	Hb101	4.8	2	8	
Figure 2d, x axis	All spp	Same data as Figure 1d-g			
Figure 2d, y axis	All spp	Same data as Figure 2c			
Figure 3a, y axis	All spp	1	1	1	(1)
Figure 3a, x axis	All spp	Same data as Figure 1d-g			
Figure 3b, y axis	All spp	4	4	4	(2)
Figure 3b, x axis	All spp	Same data as Figure 1d-g			
Figure 3c	All spp	Same data as Figure 1d-g			
Figure 3d	All spp	Same data as Figure 1d-g			
Figure 4b-c	All spp	23.4	21	24	
Figure 5 and Figure S8	DA1885 + CR266	134	134	134	
Figure S8	CR164 + CR266	139	139	139	
Figure S8	OP50 + DA1880	144	144	144	
Figure S8	Db10 + DA1880	109	109	109	

(1) Errorbars were inferred from control experiments (see Methods, section "determination of bacterial density")

(2) The 4 measurements were not fully independent (we plated 4 drops from each diluted culture, and we counted the number of colonies in each of them)

Supplementary Table 1: Sample sizes for all data shown in the main-text figures. We report the number of plates, because each plate constitutes an independent experiment. Therefore, we always performed bootstrapping on a plate-by-plate basis, rather than on a worm-by-worm basis.

Strain	Length (μm)	Diameter (μm)	Shape
DA1885	4.6 (2.5 - 8.2)	1.1 (1.0 - 1.3)	Rod
CR87	7.4 (5.5 - 10.6) *	1.7 (1.5 - 1.9)	Rods in chains
DA1880	**	1.0 (0.9 - 1.2)	Filaments
CR164	2.1 (1.3 - 3.35)	0.7 (0.6 - 0.8)	Rod
CR181	1.9 (1.1 - 3.7)	1.1 (1.0 - 1.2)	Short rod
OP50	1.8 (1.0 - 16.3)	0.6 (0.6 - 0.8)	Rod
Hb101	1.9 (1.3 - 6.9)	0.9 (0.8 - 1.0)	Rod
CR13	3.5 (2.2 - 6.0)	0.9 (0.8 - 1.1)	Rod
CR155	1.2 (0.6 - 1.9)	0.7 (0.6 - 0.8)	Short rod
CR90	2.1 (1.5 - 3.0)	1.0 (0.8 - 1.0)	Short rod
CR266	3.2 (2.1 - 5.0)	0.9 (0.7 - 1.1)	Rod
Db10	1.2 (0.9 - 1.4)	0.8 (0.6 - 0.9)	Short rod

* chain length 29.5 (11 - 48)
** filament length 14.6 (5.0 - 81.7)

Supplementary Table 2: Size distribution and cell shape of our strains. We measured the length and diameter of each of our strains (20-150 measures for each strain, for microscopy protocol see Methods). The displayed values are the median, along with the minimum and the maximum of the measures. CR87 cell chains were measured as well as individual cell length. DA1880 filament length was measured, but individual cells were not, as the filaments did not present visible septa.

Strain	Gram stain test	Aerobic growth	Nitrate reduction	Locomotion
DA1885	Gram-positive [1] or variable [3 Table 5]	Obligatory [3]	Yes [3]	variable accross genus
CR87	Gram-variable [3 Table 5]	Obligatory [3]	Yes [3]	variable accross genus
DA1880	Gram-variable or positive [3 Table 4]	Obligatory [3]	No [3]	variable accross genus
CR164	Gram-positive [1]	Obligatory [5]	No [5]	polar flagella [5]
CR181	Gram-positive [7]	Obligatory [7]	Genes present [6]	
OP50	Gram-negative [8]	Facultative [8]	Yes [20]	impaired motility [9]
Hb101	Gram-negative [8]			non-motile [21]
CR13	Gram-positive [11]		No [11]	motile [11]
CR155	Gram-negative [13]	Obligatory [13]	Yes [13]	highly motile, subpolar or peritrichous flagella) [13]
CR90	Gram-negative [15]	Obligatory [15]	Incomplete [14]	motile [15], polar flagella [19]
CR266	Gram-positive (genus) [16]	Obligatory (genus) [16]		non-motile (genus) [16]
Db10	Gram-negative [17]	Facultative [17]	Yes [17]	motile, 1-2 flagella in liquid, 10-100 flagella in solid [18]

Supplementary Table 3: Review of metabolism and locomotion in our bacterial strains, as described in the literature. Our strains were assigned a species name based on their 16S rRNA gene sequences. Data from various papers was collected to determine Gram test result, potential for anaerobic growth, nitrate reduction and motility of the corresponding species. Other metabolic information was omitted due to sparseness of information.

Strain	*Genus*	*Species*	*Alternative name*	Natural environment	Additional info
DA1885	*Bacillus*	*simplex*	*Peribacillus simplex*	some strains promote plant growth [1], surimi contaminant [4]	
CR87	*Bacillus*	*flexus*	*Priestia flexa*		
DA1880	*Bacillus*	*megaterium*	*Priestia megaterium*	plant-growth promoter [2]	
CR164	*Bacillus*	*safensis*		isolated from spacecraft surfaces [5]	
CR181	*Corynebacterium*	*variabile*	*Corynebacterium mooreparkense**	involved in cheese ripening [6]	
OP50	*Escherichia*	*coli*		enterobacteria of warm-blooded animals [10]	E. coli strains only have 20% of their genome in common [10]
Hb101	*Escherichia*	*coli*			
CR13	*Lysinibacillus*	*boronitolerans*		first isolated from soil [11], can also be found in Korean fermented soybean [12]	Contains lysine and asparate in its cell wall [12]
CR155	*Ochrobactrum*	*grignonense*	*Brucella grignonensis*	soil, wheat rhyzoplane [13]	The species name comes from the Grignon municipality, in France [13]
CR90	*Pseudomonas*	*viridiflava*	*Phytomonas viridiflava*	pathogen for a wide range of plants, can infect stems, leaves, and blossoms [15]	Green fluorescent, first isolated from sick beans in Lucerne, Switzerland [14]
CR266	*Rhodococcus*	*globerulus*			Can degrade chlorinated pollutants [16]
Db10	*Serratia*	*marcescens*		wide variety of ecological niches, opportunistic pathogen in humans [18]	

Supplementary Table 4: Additional information on our strain collection. Our strains were assigned a species identity based on their 16S rRNA gene sequences. Complete and alternative names of the identified species (alternative names include homotypic and heterotypic synonyms, and in some cases, the main name used is actually a basonym). Natural environment column contains examples of ecological niches where the corresponding species were described to grow.

Supplementary References

1. Schwartz, A. E., Ortiz, I., Maymon, M., Herbold, C.W., Fujishige, N. A., Vijanderan, J. A., Villella, W., Hanamoto, K., Diener, A., Sanders, E. R., DeMason, D. A., & Hirsc, A. M. Bacillus simplex—A Little Known PGPB with Anti-Fungal Activity—Alters Pea Legume Root Architecture and Nodule Morphology When Coinoculated with Rhizobium leguminosarum bv. viciae. *Agronomy* **3**, 595-620 (2013).
2. López-Bucio, J., Campos-Cuevas, J. C., Hernández-Calderón, E., Velásquez-Becerra, C., Farías-Rodríguez, R., Macías-Rodríguez, L. I., & Valencia-Cantero, E. Bacillus megaterium rhizobacteria promote growth and alter root-system architecture through an auxin- and ethylene-independent signaling mechanism in Arabidopsis thaliana. *Molecular plant-microbe interactions : MPMI* **20**(2), 207–217 (2007).
3. Priest, F. G., Goodfellow, M. & Todd, C. A Numerical Classification of the Genus Bacillus. *J. of General Microbiol.* **134**, 1847-1882 (1988).
4. Coton, M., Denis, C., Cadot & P., Coton, E. Biodiversity and characterization of aerobic spore-forming bacteria in surimi seafood products. *Food Microbiology* **28** (2), 252-260 (2011).
5. Satomi, M., La Duc, M. T. & Venkateswaran, K. Bacillus safensis sp. nov., isolated from spacecraft and assembly-facility surfaces. *Int. J. of Systematic and Evol. Microb.* **56**, 1735-1740 (2006).
6. Schröder, J., Maus, I., Trost, E., & Tauch, A. Complete genome sequence of Corynebacterium variabile DSM 44702 isolated from the surface of smear-ripened cheeses and insights into cheese ripening and flavor generation. *BMC genomics* **12**, 545 (2011).
7. Brennan, N. M., Brown, R., Goodfellow, M., Ward, A. C., Beresford, T. P., Simpson, P. J., Fox, P. F., & Cogan, T. M. Corynebacterium mooreparkense sp. nov. and Corynebacterium casei sp. nov., isolated from the surface of a smear-ripened cheese. *Int. J. of Systematic and Evol. Microb.* **51** (3), 843–852 (2001).
8. Tenaillon, O., Skurnik, D., Picard, B. & Denamur, E. The population genetics of commensal Escherichia coli. *Nat. Rev. Microbiol.* **8**, 207–217 (2010).
9. Arata, Y., Oshima, T., Ikeda, Y., Kimura, H., & Sako, Y. OP50, a bacterial strain conventionally used as food for laboratory maintenance of *C. elegans*, is a biofilm formation defective mutant. *microPublication biology* (2020).
10. Lukjancenko, O., Wassenaar, T. M., & Ussery, D. W. Comparison of 61 sequenced Escherichia coli genomes. *Microbial ecology* **60** (4), 708–720 (2010).
11. Ahmed, I., Yokota, A., Yamazoe, A., & Fujiwara, T. Proposal of Lysinibacillus boronitolerans gen. nov. sp. nov., and transfer of Bacillus fusiformis to Lysinibacillus fusiformis comb. nov. and Bacillus sphaericus to Lysinibacillus sphaericus comb. nov. *Int. J. of Systematic and Evol. Microb.* **57** (5), 1117–1125 (2007).
12. Nam, Y. D., Seo, M. J., Lim, S. I., & Lee, S. Y. Genome sequence of Lysinibacillus boronitolerans F1182, isolated from a traditional Korean fermented soybean product. *J. of bacteriology* **194** (21), 5988 (2012).
13. Lebuhn, M., Achouak, W., Schloter, M., Berge, O., Meier, H., Barakat, M., Hartmann, A. & Heulin, T. Taxonomic characterization of Ochrobactrum sp. isolates from soil samples and wheat roots, and description of Ochrobactrum tritici sp. nov. and Ochrobactrum grignonense sp. nov. *Int. J. of Systematic and Evol. Microb.* **50** (6), 2207 - 2223 (2000).
14. Billing, E. Pseudomonas viridiflava (Burkholder, 1930; Clara 1934). *J. Appl. Bact.* **33**, 492-500 (1970).
15. Lipps, S. M., & Samac, D. A. Pseudomonas viridiflava: An internal outsider of the Pseudomonas syringae species complex. *Mol. plant pathology* **23** (1), 3–15 (2022).
16. Bell, K. S., Philip, J. C., Aw, D. W. J. & Christofi, N. The genus Rhodococcus. *J. of Applied Microb.* **85**, 195-210 (1998).
17. Breed, R. S., Murray, E. G. D., Smith, N. R. *Bergey's Manuals of Determinative Bacteriology* (7th edition (361), The Williams & Wilkins Company, 1957).

18. Alberti, L., & Harshey, R. M. Differentiation of Serratia marcescens 274 into swimmer and swarmer cells. *J. of bacteriology* **172** (8), 4322–4328 (1990).
19. Liu, Y., Guan, X. Y., Kong, X. D., Wu, X. L, Liu, S. N., Ren, J. H., Guo, M. & Zhang Y. W. First Report of Bacterial Leaf Spot Caused by Pseudomonas viridiflava on Chinese Cabbage in China. *Plant disease* **103** (7), 1764-1764 (2019).
20. Farkas-Himsley, H., & Artman, M. Studies on nitrate reduction by Escherichia coli. *J. of bacteriology* **74** (5), 690–692 (1957).
21. Bell, C. H. The Effects of Centrifugation and Filtration as Pre-Treatments in Bacterial Retention Studies. *J. of Young Investigators* (2005).
