# Supplementary material for: Caenorhabditis elegans foraging patterns follow a simple rule of thumb
Source: Commun Biol. 2023 Aug 14;6:841. doi: 10.1038/s42003-023-05220-3 (PMC10425387; doi:10.1038/s42003-023-05220-3)
Supplement: Supplementary file 3 — Description of Additional Supplementary Files [file 42003_2023_5220_MOESM3_ESM.pdf]

Description of Additional Supplementary Files

File name: Supplementary Data 1
Description: Raw data and computer code behind the figures of the paper.
