# Supplementary figures and images for: Caenorhabditis elegans foraging patterns follow a simple rule of thumb
Source: Commun Biol. 2023 Aug 14;6:841. doi: 10.1038/s42003-023-05220-3 (PMC10425387; doi:10.1038/s42003-023-05220-3)

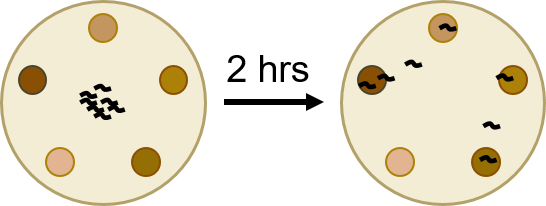

Supplement: Supplementary file 4 — Supplementary Data 1 [file 42003_2023_5220_MOESM4_ESM.zip › data_for_foraging_paper/panel_experiment.png]

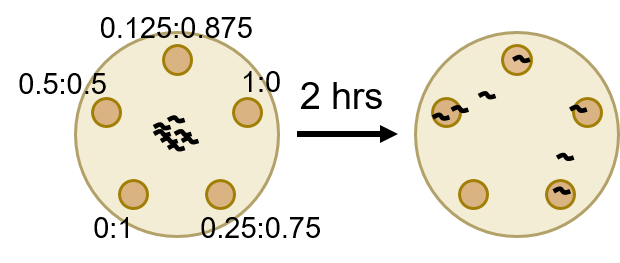

Supplement: Supplementary file 4 — Supplementary Data 1 [file 42003_2023_5220_MOESM4_ESM.zip › data_for_foraging_paper/panel_experiment_mixtures.png]

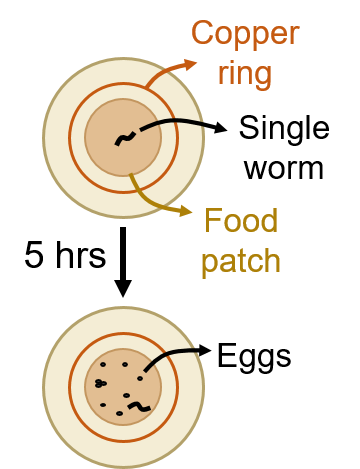

Supplement: Supplementary file 4 — Supplementary Data 1 [file 42003_2023_5220_MOESM4_ESM.zip › data_for_foraging_paper/panel_fitness.png]

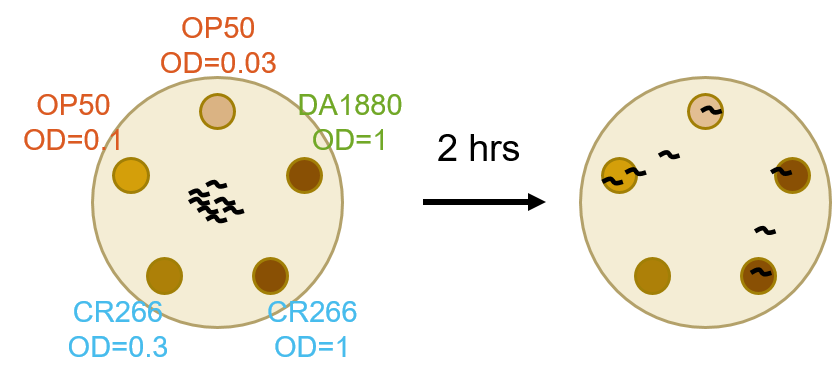

Supplement: Supplementary file 4 — Supplementary Data 1 [file 42003_2023_5220_MOESM4_ESM.zip › data_for_foraging_paper/panel_mixed_environment.png]
