# Supplementary material for: Caenorhabditis elegans foraging patterns follow a simple rule of thumb
Source: Commun Biol. 2023 Aug 14;6:841. doi: 10.1038/s42003-023-05220-3 (PMC10425387; doi:10.1038/s42003-023-05220-3)
Supplement: Supplementary file 5 — Reporting Summary [file 42003_2023_5220_MOESM5_ESM.pdf]

nature portfolio

Corresponding author(s): Alfonso Pérez Escudero

Last updated by author(s): 26/07/23

Reporting Summary

Nature Portfolio wishes to improve the reproducibility of the work that we publish. This form provides structure for consistency and transparency in reporting. For further information on Nature Portfolio policies, see our Editorial Policies and the Editorial Policy Checklist.

Statistics

For all statistical analyses, confirm that the following items are present in the figure legend, table legend, main text, or Methods section.

n/a	Confirmed	
☐	☒	The exact sample size (*n*) for each experimental group/condition, given as a discrete number and unit of measurement
☐	☒	A statement on whether measurements were taken from distinct samples or whether the same sample was measured repeatedly
☐	☒	The statistical test(s) used AND whether they are one- or two-sided<br>*Only common tests should be described solely by name; describe more complex techniques in the Methods section.*
☒	☐	A description of all covariates tested
☒	☐	A description of any assumptions or corrections, such as tests of normality and adjustment for multiple comparisons
☒	☐	A full description of the statistical parameters including central tendency (e.g. means) or other basic estimates (e.g. regression coefficient) AND variation (e.g. standard deviation) or associated estimates of uncertainty (e.g. confidence intervals)
☒	☐	For null hypothesis testing, the test statistic (e.g. *F*, *t*, *r*) with confidence intervals, effect sizes, degrees of freedom and *P* value noted<br>*Give P values as exact values whenever suitable.*
☒	☐	For Bayesian analysis, information on the choice of priors and Markov chain Monte Carlo settings
☒	☐	For hierarchical and complex designs, identification of the appropriate level for tests and full reporting of outcomes
☒	☐	Estimates of effect sizes (e.g. Cohen's *d*, Pearson's *r*), indicating how they were calculated

Our web collection on statistics for biologists contains articles on many of the points above.

Software and code

Policy information about availability of computer code

Data collection	Custom Python routines using the Opentrons API, v1.0, to control the Opentrons OT2 robot
Data analysis	Custom code written in Matlab R2019a to analyze images from the scanners and to perform the data analysis and the figures.

For manuscripts utilizing custom algorithms or software that are central to the research but not yet described in published literature, software must be made available to editors and reviewers. We strongly encourage code deposition in a community repository (e.g. GitHub). See the Nature Portfolio guidelines for submitting code & software for further information.

Data

Policy information about availability of data

All manuscripts must include a data availability statement. This statement should provide the following information, where applicable:

- Accession codes, unique identifiers, or web links for publicly available datasets
- A description of any restrictions on data availability
- For clinical datasets or third party data, please ensure that the statement adheres to our policy

Our manuscrip does not contain standardized data that should be deposited in a repository. All data and analysis computer code is in the Supplementary Dataset 1.

Human research participants

Policy information about studies involving human research participants and Sex and Gender in Research.

Reporting on sex and gender	*Use the terms sex (biological attribute) and gender (shaped by social and cultural circumstances) carefully in order to avoid confusing both terms. Indicate if findings apply to only one sex or gender; describe whether sex and gender were considered in study design whether sex and/or gender was determined based on self-reporting or assigned and methods used. Provide in the source data disaggregated sex and gender data where this information has been collected, and consent has been obtained for sharing of individual-level data; provide overall numbers in this Reporting Summary. Please state if this information has not been collected. Report sex- and gender-based analyses where performed, justify reasons for lack of sex- and gender-based analysis.*
Population characteristics	*Describe the covariate-relevant population characteristics of the human research participants (e.g. age, genotypic information, past and current diagnosis and treatment categories). If you filled out the behavioural & social sciences study design questions and have nothing to add here, write "See above."*
Recruitment	*Describe how participants were recruited. Outline any potential self-selection bias or other biases that may be present and how these are likely to impact results.*
Ethics oversight	*Identify the organization(s) that approved the study protocol.*

Note that full information on the approval of the study protocol must also be provided in the manuscript.

Field-specific reporting

Please select the one below that is the best fit for your research. If you are not sure, read the appropriate sections before making your selection.

☒ Life sciences ☐ Behavioural & social sciences ☐ Ecological, evolutionary & environmental sciences

For a reference copy of the document with all sections, see nature.com/documents/nr-reporting-summary-flat.pdf

Life sciences study design

All studies must disclose on these points even when the disclosure is negative.

Sample size	Sample sizes were determined via preliminary experiments, by visual inspection of the resulting errorbars. Our conclusions depend only weakly on any statistical test, so we did not use any statistical method to determine sample size.
Data exclusions	The article presents results from three separate experimental sessions. No data from any of these sessions was excluded. Preliminary experiments are not presented in the paper, and were consistent with the results we present except for one case where we had clear contamination in one of our bacterial cultures (optical density was shifted to values atypical for the desired species).
Replication	The paper presents data collected with a 2-year separation, and in two different laboratories (Figure S2).
Randomization	Samples were randomized using Python's random number generator, so that different conditions were prepared at random times during the experiment. Individuals were pipetted from an eppendorf into the plates, mixing thoroughly every time to pick a random set of individuals.
Blinding	Experiments were performed in a semi-blind way: Conditions were prepared and randomized the previous day, and labeled with a binary number. While experimenters could in theory read the number and look for the condition, in practice the table with conditions was buried in computer code, and the experimenters never looked at it. Most data were collected and analyzed automatically. We performed manual confirmation of number of worms in doubtful images, and this process was blind for the human operator (they did not know what condition they were handling in each time, nor where in the plate were the worms they were seeing).

Reporting for specific materials, systems and methods

We require information from authors about some types of materials, experimental systems and methods used in many studies. Here, indicate whether each material, system or method listed is relevant to your study. If you are not sure if a list item applies to your research, read the appropriate section before selecting a response.

Materials & experimental systems

n/a	Involved in the study
☒	☐ Antibodies
☒	☐ Eukaryotic cell lines
☒	☐ Palaeontology and archaeology
☐	☒ Animals and other organisms
☒	☐ Clinical data
☒	☐ Dual use research of concern

Methods

n/a	Involved in the study
☒	☐ ChIP-seq
☒	☐ Flow cytometry
☒	☐ MRI-based neuroimaging

Animals and other research organisms

Policy information about studies involving animals; ARRIVE guidelines recommended for reporting animal research, and Sex and Gender in Research

Laboratory animals	Caenorhabditis elegans, strain N2, 48-hour old
Wild animals	No
Reporting on sex	In our culture conditions, 99% of worms are hermaphrodites. We did not monitor or control for sex composition of our population.
Field-collected samples	*For laboratory work with field-collected samples, describe all relevant parameters such as housing, maintenance, temperature, photoperiod and end-of-experiment protocol OR state that the study did not involve samples collected from the field.*
Ethics oversight	Experiments with Caenorhabditis elegans do not require ethical approval in Europe.

Note that full information on the approval of the study protocol must also be provided in the manuscript.
